# Memory reconsolidation may be disrupted by a distractor stimulus presented during reactivation

**DOI:** 10.1038/srep13633

**Published:** 2015-09-02

**Authors:** Ana Paula Crestani, Flávia Zacouteguy Boos, Josué Haubrich, Rodrigo Ordoñez Sierra, Fabiana Santana, Johanna Marcela Duran Molina, Lindsey de Freitas Cassini, Lucas de Oliveira Alvares, Jorge Alberto Quillfeldt

**Affiliations:** 1 Psychobiology and Neurocomputation Lab, Federal University of Rio Grande do Sul, Porto Alegre, Brazil; 2 Neurosciences Graduate Program, Federal University of Rio Grande do Sul, Porto Alegre, Brazil

## Abstract

Memories can be destabilized by the reexposure to the training context, and may reconsolidate into a modified engram. Reconsolidation relies on some particular molecular mechanisms involving LVGCCs and GluN2B-containing NMDARs. In this study we investigate the interference caused by the presence of a distractor - a brief, unanticipated stimulus that impair a fear memory expression - during the reactivation session, and tested the hypothesis that this disruptive effect relies on a reconsolidation process. Rats previously trained in the contextual fear conditioning (CFC) were reactivated in the presence or absence of a distractor stimulus. In the test, groups reactivated in the original context with distractor displayed a reduction of the freezing response lasting up to 20 days. To check for the involvement of destabilization / reconsolidation mechanisms, we studied the effect of systemic nimodipine (a L-VGCC blocker) or intra-CA1 ifenprodil (a selective GluN2B/NMDAR antagonist) infused right before the reactivation session. Both treatments were able to prevent the disruptive effect of distraction. Ifenprodil results also bolstered the case for hippocampus as the putative brain structure hosting this phenomenon. Our results provide some evidence in support of a behavioral, non-invasive procedure that was able to disrupt an aversive memory in a long-lasting way.

A considerable amount of evidence has shown that consolidated memories can enter into a new labile state after reactivation, requiring a later restabilization process known as reconsolidation^1^. Specific molecular mechanisms were identified that trigger memory trace destabilization, including Ca^++^ influx mediated by Voltage Gated Calcium (VGCCs) or NMDA channels^2^,^3^,^4^, the action of CB1 receptors^5^, and protein degradation dependent on the ubiquitin/proteasome system^6^,^7^,^8^. Even in a labile state, memories remain susceptible to pharmacological and behavioral disruption^9^,^10^,^11^,^12^,^13^,^14^,^15^,^16^,^17^,^18^,^19^,^20^. The underlying hypothesis is that labilization-reconsolidation processes may enable the modification of pre-existing memories. To the extent that this is true, disrupting memory reconsolidation is expected to have an impact as a treatment strategy concerning fear-related memories.

“Distraction” – a concept that usually belongs to the realm of attentional studies – can be operationally defined here as a short-term, irrelevant and unanticipated stimulus capable of *displacing* the representation of a concomitant conditioning factor (context, shock, appetitive reward, etc) from working memory^21^,^22^. The resulting decay of the memory trace could take place without necessarily incorporating any distractor details as additional information^23^. In support of this idea, human studies have shown that distractor stimuli may impair unwanted memories in some specific situations^24^,^25^,^26^,^27^,^28^,^29^,^30^. Consistent with that, previous work from our lab where a fear conditiong memory is reactivated by pairing context with a highly palatable appetitive stimulus (chocolate), was able to labilize the aversive memory trace, modifying its emotional valence^4^. It is our hypothesis that, in these cases, distraction interferes with memory by disturbing the process known as reconsolidation.

In order to test if distractor may effectively disrupt fear memory reconsolidation, the stimuli were presented during the reactivation session and memory retention was measured. Additionally, we investigated if the observed effects actually rely on the well-described destabilization/reconsolidation neurochemical mechanisms in order to confirm their nature.

## Material and Methods

### Animals

Male Wistar rats weighting 250–350 g from our University breeding colony (CREAL/UFRGS) were used. Animals were housed in plastic cages, four to five in a cage, under a 12 h light/dark cycle and at constant temperature of 24 ± 1 °C, with water and food *ad libitum*. All experiments were conducted in accordance to local animal care guidelines (Brazilian Federal Law 11,794/2008) and approved by the Ethics in the Use of Experimental Animals Committee of Federal University of Rio Grande do Sul (CEUA, Project UFRGS #23,326).

### Behavioral procedure

Each experiment consisted of three or four phases – conditioning, reactivation, test and retest sessions – as described below (see also the diagrams in figure). Memory was measured quantifying freezing behavior: *freezing* is defined as the absence of all movements except those related to breathing, and expressed as percentage of the total session time.

### Conditioning chamber (context)

The conditioning chamber (context training) consisted of an illuminated Plexiglas box (20 × 25 × 22 cm, with a grid of parallel 0.1 cm caliber stainless steel bars spaced 1.0 cm apart) with constant fan background sound (white noise). The novel context was a rectangular box with dimensions similar to the conditioning one, with a smooth floor, one wall painted with black-and-white vertical stripes, and without background white noise.

### Contextual Fear Conditioning (CFC): training session

In the training session, rats were placed in the conditioning chamber to habituate for 3 min before receiving two 2-s, 0.7-mA footshocks separated by a 30-s interval (unconditioned stimulus); they were kept in the conditioning environment for additional 30-s before returning to their homecages.

### Reactivation session: distractor stimulus presentation

Two days after training, rats were either re-exposed to the training context (reactivation session), or exposed to a novel context, for 5 min. The unconditioned stimulus (US) – footshock – was absent either during the reactivation session in the original and the novel context exposition: after the first minute of the reactivation session, a *distractor stimulus* (DIST) – a soft air puff from a wash bottle – was directed to the animal´s head and torso every time the animal expressed the freezing response.

### Test session

Test consisted of measuring animal´s freezing response to a 4 min exposition to the same training context on day 5.

### Reinstatement session

24 hours after test, subjects received two successive, non-paired footshocks of 0.3 mA (weak), each with 2 sec duration, in a 15 × 10 × 11.5 cm blue polypropylene box; they were retested (CFC training context) for reinstatement in the following day.

### Retest session

Animals were re-tested for 4 min in the same training context on day 20.

### Stereotaxic surgery and cannulae placement

Animals were anesthetized with a ketamine and xylazine association (75 and 10 mg/kg, respectively) infused intraperitoneally. A 22-gauge guide cannulae was implanted bilaterally at AP = −4.0 mm (from Bregma), LL = ±3.0 mm, DV = −1.6 mm, positioned just 1.0 mm above the CA1 area of the dorsal hippocampus (according to Paxinos & Watson, 1998)^31^. After a recovery from the surgery of at least 5 days, behavioral procedures were performed. After that, all animals were sacrificed, their brains dissected and fixed on 10% formaldehyde in order to verify the cannulae placement under low magnification. Animals with inaccurate cannulae placements were excluded from the statistical analysis.

### Drugs

Nimodipine (Sigma), an antagonist of the L-type voltage-gated calcium channels (LVGCCs) was dissolved in sterile isotonic saline solution with 8% dimethylsulfoxide to a concentration of 16 mg/mL. Nimodipine or its vehicle were injected systemically (subcutaneously - s.c.) 30 min before the reactivation session, both to a total volume of 1 mL/kg. Ifenprodil (Sigma), the selective antagonist of the GluN2B subunit of NMDAR, was dissolved in a phosphate-buffered saline solution to a concentration of 1 mg/mL and it, or its vehicle, was infused intrahippocampally (in CA1) at a slow rate (20 μL/h; 0.5 μL/side), 15 min prior to the reactivation session.

### Statistical analysis

After confirming the homocedasticity (Levene test) and normality of the data distribution (Kolmogorov-Smirnov test), experiments were analyzed by ANOVA for Repeated Measures followed by a Newman-Keuls *post hoc* test, when applicable. Significance was set at *P *< 0.05.

## Results

### Memory reactivation in the presence of distractor stimulus leads to an enduring fear memory impairment

**Figure 1:**
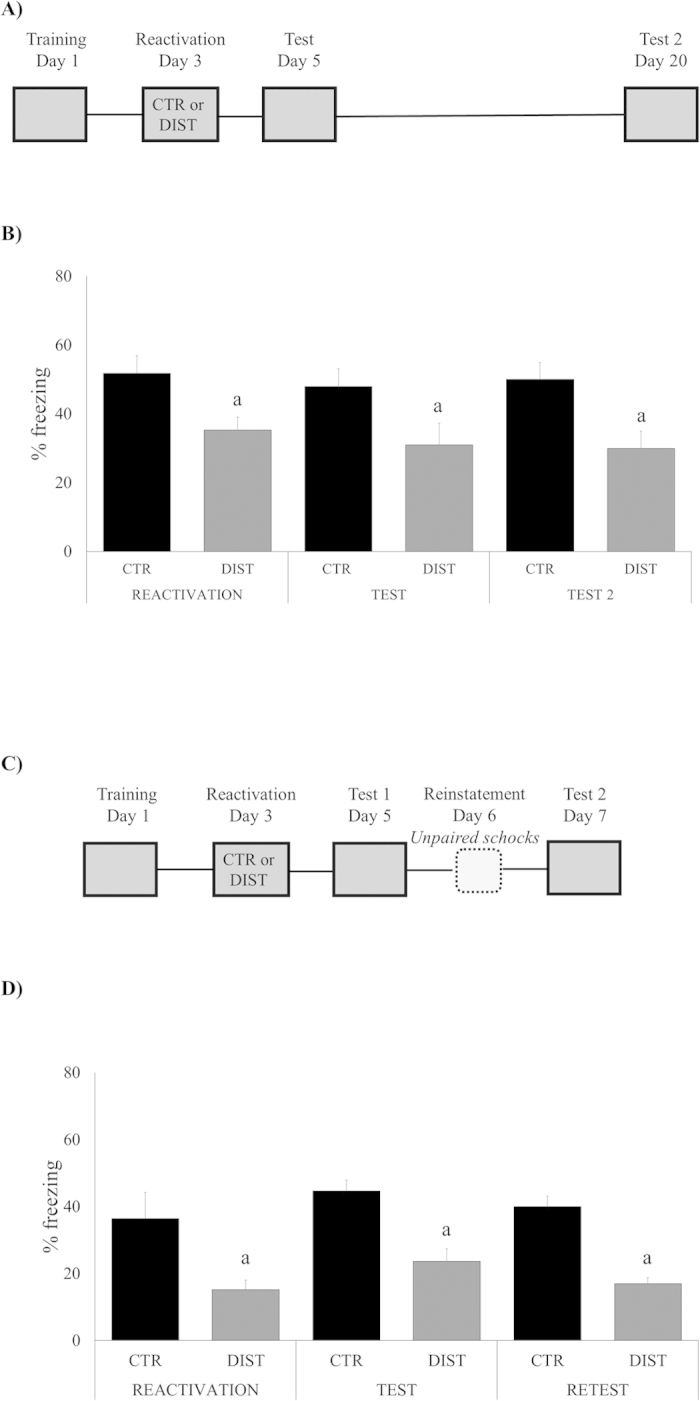
Memory reactivation in the presence of a distractor disrupts fear memory expression in a long-lasting way. (**A**) Schematic representation of the behavioral procedures: rats were *re-exposed to the fear conditioning context without the US (shock)* 48 h after training (reactivation session); all groups were *tested* on day 5, and *retested* on day 20, *in this same context*. (**B**) Percent of freezing time during reactivation, test and retest sessions expressed as mean ± S.E.M. (CTR – Controls, N = 12 or DIST – Distractor, N = 11). (a) significantly different from respective control group (*P *< 0.05; effect of groups, ANOVA for Repeated Measures). (**C)** Schematic representation of the behavioral procedures: rats were *re-exposed to the fear conditioning context without the US (shock)* 48h after training (reactivation session); all groups were *tested* on day 5, *exposed to unconditioned stimulus* (reinstatement session) on day 6, and *retest* on day 7. (**D)** Percent of freezing time during reactivation, test and retest sessions expressed as mean ± S.E.M. (CTR – Controls, N = 10 or DIST – Distractor, N = 8). (a) significantly different from respective control group (*P *< 0.05; effect of groups, ANOVA for Repeated Measures).

In our first experiment, we evaluate whether distractor stimulus was able to disrupt memory reconsolidation – a labile state that follows the reactivation that makes it susceptible to disruption (Nader *et al*., 2009). Rats were trained in the contextual fear conditioning (CFC) and later re-exposed to the same context (reactivation) in order to induce memory destabilization. In the *distracted group*, a soft air puff (the distractor stimulus) was applied to the animals during their freezing, while in the control group, reactivation did not involve any distractor stimulus. Animals were tested at 2 and retested at 20 days after training, in the same context (Fig. 1A,B). ANOVA for Repeated Measures revealed a significant effect of *group* (F_(1,21) _= 9.3929, *P *= 0.0059), but not of session (F_(1,21) _= 0.5431, *P *= 0.5850) or group*session interaction (F_(1,21) _= 0.0105, *P *= 0.9896): notice that DIST group exhibited less freezing than its control along all sessions. Additionally, other animals were retest after exposure to the unconditioned stimulus, i.e. *reinstatement* procedure (Fig. 1C,D). ANOVA for Repeated Measures showed a significant effect of *group* (F_(1,16) _= 29.4724, *P *= 0,0001), but not of session (F_(1,16) _= 1.9626, *P *= 0.15704) or group*session interaction (F_(1,16) _= 0.0324, *P *= 0.9682): demonstrating that freezing levels of DIST group was maintained in lower levels when compared to control during all sessions, even after unconditioned stimulus.

### Disruption by the distractor stimulus only takes place if animals are re-exposed to the original training context

**Figure 2:**
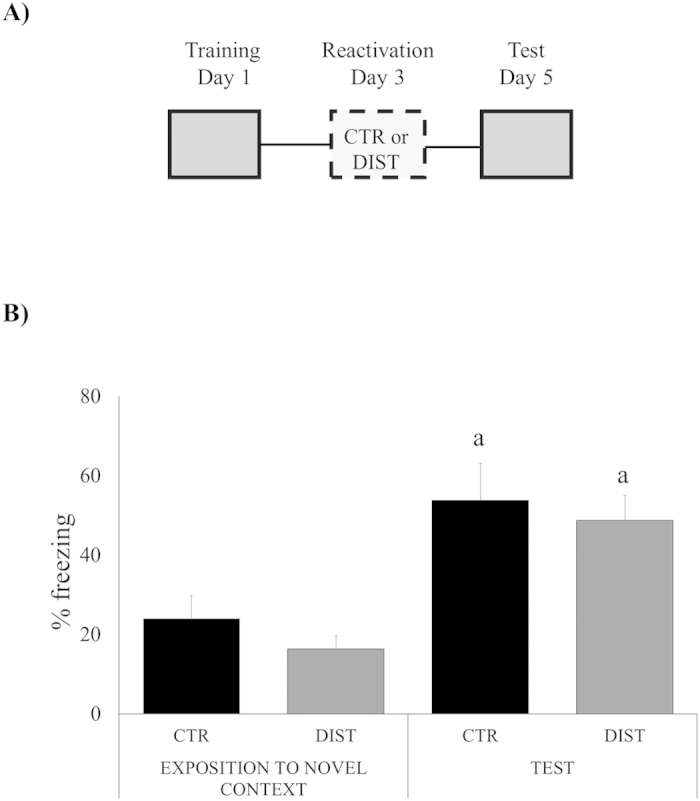
Memory reactivation does not take place when reactivation with distractor stimulus is tried in a novel context. (**A)** Schematic representation of the behavioral procedures: rats were *exposed to a novel context without the US (shock)* 48 h after training reactivation session); on day 5, all groups were *tested in the original training context*. (**B)** Percent of freezing time during exposition to a novel context and test sessions expressed as mean ± S.E.M. (CTR – Controls, N = 7 or DIST – Distractor, N = 8). (a) both groups, CTR and DIST differ significantly only between sessions (*P *< 0.05; effect of sessions, ANOVA for Repeated Measures).

Reconsolidation takes place only when reactivation is performed in the exact training context (see, e.g., Winocur *et al*., 2009), thus it is important to evaluate the context-dependency of the distractor effect. Two groups of rats (distracted and control) were exposed to a novel, different context, after which they were tested in the original training context (Fig. 2). ANOVA for Repeated Measures has shown significant effect of *session* (F_(1,13) _= 107.7779, *P *= 0,0000), but not of group (F_(1,13) _= 4.2672, *P *= 0.0611) or group*session interaction (F_(1,13) _= 0.1271, *P *= 0.7277), with no difference between groups in each session (only along them).

### The distractor-induced memory disruption involves trace destabilization mediated by L-type voltage-gated Ca^++^ channels

**Figure 3:**
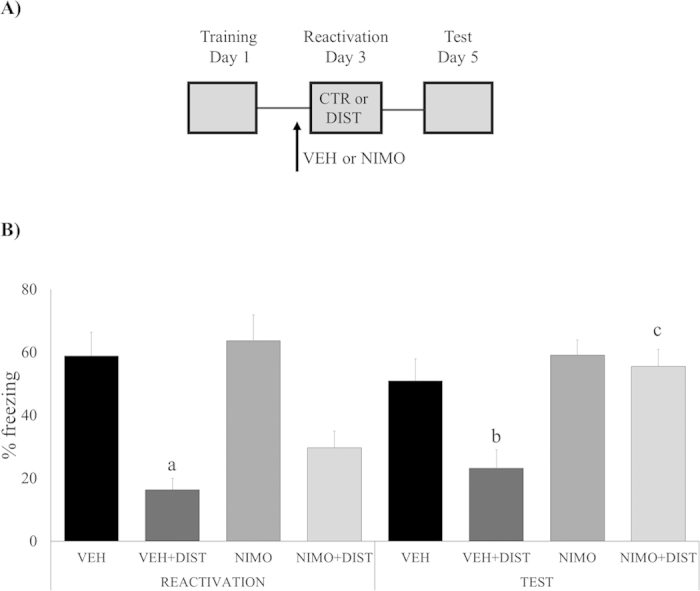
Memory disruption is dependent on destabilization mediated by L-type voltage-gated Ca++ channels. (**A**) Schematic representation of the behavioral procedures: 48 h after training, rats received a s.c. infusion of nimodipine (NIMO) or its vehicle (VEH), and, 30 min later, were *re-exposed to the fear conditioning context without the US (shock) –* the Reactivation session – either *with or without the presence of a distractor* (DIST); all groups were *tested* on day 5. (**B**) Percent of freezing time during Reactivation and Test sessions expressed as mean ± S.E.M. (VEH, N = 8, VEH + DIST, N = 9, NIMO, N = 8, and NIMO + DIST, N = 8). (a) significantly different from both control groups, VEH and NIMO (*P *< 0.05; Newman-Keuls post-hoc test); (b) significantly different from all the other groups (*P *< 0.05; Newman-Keuls post-hoc test); (c) performance differs significantly between sessions (*P *< 0.05; Newman-Keuls post-hoc test).

One necessary step in the reactivation/reconsolidation process is trace destabilization, which is mediated by L-type voltage-gated Ca^++^ channels: nimodipine, a selective LVGCC antagonist, can prevent it without affecting concomitant processes such as memory storage or retrieval (de Oliveira Alvares *et al.*, 2013; Suzuki *et al*., 2008; Sierra *et al*., 2013; Cassini *et al*., 2013). Accordingly, if memory disruption caused by the distractor stimulus actually relies on a memory reconsolidation process, then nimodipine should prevent the effect (Fig. 3). ANOVA for Repeated Measures has shown a significant effect of group (F_(3,29) _= 28.3694 , *P *= 0.0000), drug (F_(3,29) _= 8.3980, *P *= 0.0071) and session*group interaction (F_(3,29) _= 11.7974, *P *= 0.0019), but not of session (F_(3,29) _= 2.3873, *P *= 0.1332), and the remaining interactions: session*drug (F_(3,29) _= 2.9146, *P *= 0.0985) and session*group*drug (F_(3,29) _= 1.4041, *P *= 0.2457). In the *test* session, Newman-Keuls *post-hoc* analysis has shown that only the VEH + DIST group expressed lower freezing levels compared to all the other groups, VEH (*P *= 0.02757), NIMO (*P *= 0.01535), or NIMO + DIST (*P *= 0.0168). In the *reactivation* session, however, both VEH + DIST and NIMO + DIST groups have shown lower freezing levels compared to the other groups, VEH (*P *= 0.0030 and 0.0348, respectively) and NIMO (*P *= 0.0015 and 0.0243, respectively (Newman-Keuls *post-hoc* analysis). The only group to present a significantly different performance *between sessions* was the NIMO + DIST one (*P *= 0.0014; Newman-Keuls post-hoc test).

### Dependency on GluN2B-containing NMDA receptors in the hippocampus also point to a trace destabilization mechanism behind the observed disruption effect

**Figure 4:**
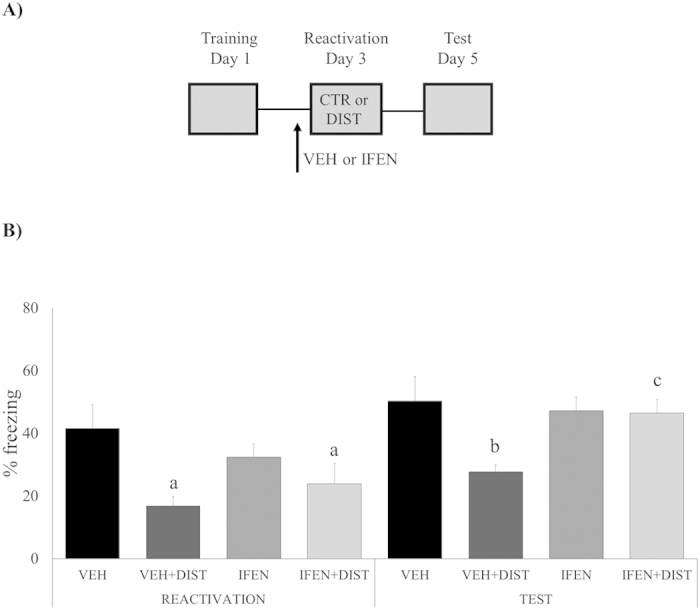
Memory disruption is dependent on destabilization mediated by GluN2B-containing NMDA receptors in the hippocampus. (**A)** Schematic representation of the behavioral procedures: 48 h after training, rats received an intrahippocampal (CA1 area) infusion of ifenprodil (IFEN) or its vehicle (VEH), and, 15 min later, were *re-exposed to the fear conditioning context without the US (shock) –* the Reactivation session – either *with or without the presence of a distractor* (DIST); all groups were *tested* on day 5. (**B**) Percent of freezing time during Reactivation and Test sessions expressed as mean ± S.E.M. (VEH, N = 9, VEH + DIST, N = 13, IFEN, N = 12, and IFEN + DIST, N = 12). (a) significantly different from its control group, VEH (*P *< 0.05; Newman-Keuls post-hoc test); (b) significantly different from its control group, VEH (*P *< 0.05; Newman-Keuls post-hoc test); in both sessions, there is no significant difference among the remaining (unmarked) groups; (c) performance differs significantly between sessions (*P *< 0.05; Newman-Keuls post-hoc test).

Trace destabilization was also shown to depend on NMDA receptors containing the GluN2B subunit (Ben Mamou *et al*., 2006; Milton *et al*., 2013; Haubrich *et al*., 2015), particularly in the hippocampus, a brain structure involved in contextual fear memory reconsolidation (Anagnostaras *et al*., 2001; Rudy, *et al*., 2004; Winocur *et al*., 2009). Thus, we infused the selective GluN2B antagonist ifenprodil bilaterally into the CA1 hippocampal area 15 min prior to reactivation session aiming to prevent memory destabilization, and, thus, gather support for the reconsolidation hypothesis (Fig. 4). ANOVA for Repeated Measures has shown a significant effect of group (F_(3,42) _= 17.0813, *P *= 0.0002), session (F_(3,42) _= 26.1011, *P *= 0.0000) and group*drug interaction (F_(3,42) _= 8.5353, *P *= 0.0056), but not of drug (F_(3,42) _= 0.2191, *P *= 0.6421), or the following interactions: session*group (F_(3,42) _= 1.6320, *P *= 0.2084), session*drug (F_(3,42) _= 4.0118, *P *= 0.0517) and session*group*drug (F_(3,42) _= 0.0454, *P *= 0.8323). In the *test* session, Newman-Keuls *post-hoc* analysis has shown that the VEH + DIST group expressed lower freezing levels compared to the VEH (*P *= 0.0326), but not to the remaining groups (*P *= 0.0987 and 0.0815, respectively). In the *reactivation* session, Newman-Keuls *post-hoc* analysis revealed that both VEH + DIST and IFEN + DIST groups were significantly smaller than the VEH control (*P *= 0.0032 and 0.0248, respectively), that, on its turn, was similar to IFEN (*P *= 0.5072). Similar to the nimodipine experiment, IFEN + DIST was the only group to present a significantly different performance *between sessions* (*P *= 0.0010; Newman-Keuls post-hoc test).

## Discussion

In the present study, we have investigated the disruptive effect of a distractor stimulus during the reconsolidation of the original fear memory, with its consequent impairment. In Fig. 1, we observe that the distractor stimulus was able to reduce the freezing to the lower levels observed in the test session. Perseverance of this modified response for almost 3 weeks (see test 2, Fig. 1B) supports the idea that the modified engram did not undergo anything resembling a “spontaneous recovery”: would this be the case, we could suspect of an extinction effect. Considering also that the effect was not sensitive to a reinstatement protocol (Fig. 1C,D) and the fact that the reactivation session was too short to extinguish any memory trace, our results clearly refute this possibility. All things considered, we interpret the observed fear memory change as the result of a reconsolidation process.

Consistently, Fig. 2 shows that the distractor effect requires the reexposition to the very same training context in order to be reactivated, i.e., destabilized/reconsolidated. That is why the lower freezing observed in the *exposition to the novel context* session (Fig. 2B) was only expected.

Notice that the significant reduction of % freezing *during* the reactivation session, as observed in our experiments (see “reactivation” in Figs 1,3 and 4) is, in itself, a trivial and expected effect, since reaction to the airpuff stimulus naturally competes with freezing behavior “stealing” some of its expression time.

To this point, however, our behavioral results do not prove that the distraction-induced effect was actually mediated by a reconsolidation phenomenon, so it was necessary to investigate some underlying mechanisms in order to support that conclusion. It is accepted by many authors that trace destabilization is a necessary step in the reactivation/reconsolidation process, and blocking its neurochemical substrates should be a robust way of testing for the hypothesis of the presence of a reconsolidation process. Since L-type Voltage-Gated Calcium Channels were shown to mediate destabilization without affecting concomitant processes such as memory storage or retrieval^3^,^5^,^12^,^32^ we injected (systemically) the selective antagonist nimodipine. Accordingly, if memory disruption caused by the distractor stimulus actually relies on a reconsolidation process, then nimodipine should prevent the effect, exactly as we have found (Fig. 3). As trace destabilization is likewise dependent on GluN2B-containing NMDA receptors^2^,^4^,^33^, and since this also takes place in the hippocampus, an area of paramount importance for memory reconsolidation^34^,^35^,^36^, we infused ifenprodil into CA1 before the reactivation session, having found the same prevention effect observed with systemic nimodipine (Fig. 4). In both experiments, memory destabilization was prevented, and, as a consequence, the distractor stimulus failed to disrupt fear memory (see Figs 3 and 4): these findings are fully consistent with the nature of a reconsolidation process. It is important to notice that both destabilization blockers were infused in concentrations that do not affect retrieval *per se*, in order to be useful to check for each specific dependence – upon L-VGCCs or GluN2B-NMDARs, respectively – by reverting the distractor effect. Ifenprodil results also bolstered the case for hippocampus as the putative brain structure hosting the phenomenon of memory reconsolidation.

The fact that performance of the distracted groups resulted in the reduction of the response naturally prompts the question of whether this was, or not, a simple case of “facilitation of extinction”. Reconsolidation and extinction have some markedly different properties: while the first seems to modify the engram in a more permanent way^11^,^37^,^38^ – either reducing or increasing its intensity or quality^3^, the second usually has only a transient inhibitory effect upon memory, usually involving a new, second trace^39^,^40^,^41^,^42^,^43^,^44^. In extinction, fear response may return through several routes, either by the passage of time (spontaneous recovery) or after exposure to the unconditioned stimulus (reinstatement), for instance^39^,^45^,^46^,^47^. Among other reasons, we believe our effect cannot be considered an extinction because the reexposure duration is too short to result in extinction. We have extensively studied and discussed this elsewhere^3^,^4^,^48^, but, in particular, we have shown that reactivation sessions lasting from 3 to 9 minutes may result in a protein synthesis-dependent reconsolidation process, while requiring a context re-exposure of at least 25 to 30 minutes^4^,^48^ to induce a true extinction. Thus, not only the 5-min re-exposure/reactivation session (with or without a distractor) lays precisely in the time window typical for reconsolidation induction, but the observed effect also does not involve neither reinstatement, nor spontaneous recovery, which would otherwise characterize an extinction process.

In this work, “distraction” was operationally defined as a short interference able to expel relevant contextual information from working memory^21^,^22^, enough to interfere with long-term memory formation. It does not imply, automatically, in the incorporation of any distractor information *per se* into the memory trace^23^. However, our results cannot discard completely the possibility that new information was inserted into the fear engram in order to reduce the aversive valence of the original memory: this would be interference by addition, not displacement, i.e., a form of *updating*, yet another possible reconsolidation outcome^3^,^17^,^37^. Indeed, we have previously demonstrated that the memory valence might be effectively altered by pairing a positive appetitive stimulus (chocolate in that case) with context-re-exposure^4^.

That reconsolidation can be pharmacologically disrupted is a well established fact^1^,^49^,^50^,^51^,^52^,^53^,^54^. Compared to this, its hard to overrate the clinical relevance of the availability of a behavioral, essentially non-invasive procedure able to modify the emotional valence of an aversive memory. There is an extensive literature on the beneficial effects of distractor stimuli upon unwanted memories in humans^24^,^25^,^26^,^29^,^30^, including Cognitive Behavioral Therapy techniques such as EMDR - Eye Movement Desensitization and Reprocessing^27^,^28^, but their mechanisms are still poorly understood. In EMDR, “reprocessing” was indeed suggested to involve reconsolidation to some extent^28^. In agreement with previous reports^11^,^55^,^56^, our results have shown that an aversive memory may be effectively modified by a drug-free approach – distractor stimulus presented during reactivation in a controlled context – able to modify the emotional valence of an aversive memory in a relatively long-lasting way.

## Additional Information

**How to cite this article**: Crestani, A. P. *et al*. Memory reconsolidation may be disrupted by a distractor stimulus presented during reactivation. *Sci. Rep*. **5**, 13633; doi: 10.1038/srep13633 (2015).
